# First Report on the *Streptococcus* *gallolyticus* (*S. bovis* Biotype I) DSM 13808 Exopolysaccharide Structure

**DOI:** 10.3390/ijms231911797

**Published:** 2022-10-05

**Authors:** Anna Maciejewska, Czeslaw Lugowski, Jolanta Lukasiewicz

**Affiliations:** Laboratory of Microbial Immunochemistry and Vaccines, Ludwik Hirszfeld Institute of Immunology and Experimental Therapy, Polish Academy of Sciences, Weigla 12, 53-114 Wroclaw, Poland

**Keywords:** exopolysaccharide, EPS, NMR, mass spectrometry, *Streptococcus gallolyticus*, *Streptococcus bovis* (biotype I)

## Abstract

*Streptococcus gallolyticus* subspecies *gallolyticus*, known as *Streptococcus bovis* biotype I, is a facultative pathogen causing bacteraemia, infective endocarditis and sepsis that has been linked with colorectal cancer (CRC), but this correlation is still unclear. Bacterial surface structures, such as the major sugar antigens exposed to the outside of the microorganism, are potential virulence factors. One of the primary sugar antigens loosely attached to the cell surface is the biofilm component, exopolysaccharide (EPS). EPSs of *S. bovis* are poorly characterized molecules. Until now, only one *S. macedonicus* Sc136 EPS structure was known to the entire *S. bovis* group. The *S. gallolyticus* DSM 13808 EPS was investigated by chemical analysis, mass spectrometry and nuclear magnetic resonance (NMR) spectroscopy. The hexasaccharide repeating unit of the EPS, containing four Glc, two Rha residues and one phosphate group, has been described “ →6)-α-d-Glc*p*-(1→3)-β-l-Rha*p*-(1→4)-β-d-Glc*p*-(1→3)-[β-d-Glc*p*-(1→2)]-α-l-Rha*p*-(1→2)-α-d-Glc*p*-(1→P→”.

## 1. Introduction

Association of infections caused by specific pathogens with neoplasms development is currently interesting and a promising research trend. Evidence for a connection between microorganisms and diseases, e.g., the human papillomavirus (HPV) responsible for cervical cancer development [1], or *Helicobacter pylori*, which is a known risk factor for gastric cancer of the intestinal type [2], has been found. Another example is *Streptococcus bovis*—a facultative pathogen causing bacteraemia, endocarditis and sepsis that has been linked with colorectal cancer (CRC) since the 1950s, but this correlation is still unclear. Worldwide, CRC is one of the major medical problems. It ranks as the third most common malignant neoplastic disease after breast and lung cancer in women and prostate and lung cancer in men [3,4]. Following lung cancer, CRC is the second highest cause of global cancer mortality, with an estimated 9.4% of cancer deaths in 2020 [5].

*S. bovis* is a Gram-positive bacterium and a gastrointestinal commensal found in animals and 16% of healthy people [6]. The most recent taxonomy identified the following species and subspecies among *S. bovis*: *S. gallolyticus* divided into subspecies *gallolyticus* (biotype I), *pasteurianus* (biotype II/2), *macedonicus* and *S. infantarius* with the subspecies *infantarius* (biotype II/1) and *coli* (also called *lutetiensis*) (biotype II/1) [7]. Due to the medical aspects, the greatest interest of researchers is focused on *S. gallolyticus* subspecies *gallolyticus* (Sgg) as 24% of streptococcal endocarditis is caused by Sgg. It has been shown that 65% of patients with infections caused by these bacteria are diagnosed with colorectal cancer [8]. Butt et al. demonstrated that risk of CRC is increased in individuals with serum antibody response to Sgg pilus protein [9]. To date, little is known about factors determining the relationship between CRC and *S. bovis*. *In vitro* and *in vivo* mouse models [10] demonstrated that *S. gallolyticus* actively promotes proliferation of human colon cancer cells depending on the growth phase of bacteria, direct contact of bacterial antigens with cells and from β-catenin (a key host molecule in the development of CRC). Not all CRC cell lines were sensitive to Sgg, suggesting that host traits may also have importance. Bacterial surface structures, such as piluses (Pil1, Pil2, Pil3), or the major sugar antigens exposed to the outside of the microorganism are potential virulence factors of Sgg [11]. Boleij et al. [12] hypothesized polysaccharide capsule- and piluslike structures as the main determinants of Sgg-specific association with endocarditis and CRC. The authors suggested the importance of these factors in observed low adhesiveness, inability to internalize epithelial cells by Sgg and biofilm formation on collagen-rich surfaces. Recently, Taylor et al. showed that the type VII secretion system (SggT7SST05), being important in bacterial virulence and persistent infection, is significant for Sgg adherence to CRC cells and stimulation of CRC cell proliferation [13].

As polysaccharide surface antigens were indicated among potential factors involved in association between Sgg and CRC, it is important to investigate sugar antigens characteristic of Sgg. One of the major sugar antigens loosely attached to the cell surface is exopolysaccharide (EPS). Being a long-chain homopolymer or heteropolymer composed of oligosaccharide repeating units is essential for protective function against environmental and biological factors. It plays an important role in forming of biofilm and enabling life in mono- or multi-species communities. 

Knowledge regarding the composition, architecture and structure of the bacterial cell envelope in *S. bovis* group is inadequate. It is known that *S. bovis* produces capsules containing a large amount of α-d-glucose polymers [14] and lipoteichoic acid, but the complete structure of EPS for this bacterium is known only for one strain: *S. macedonicus* Sc136 [15].

Therefore, the aim of this study was to extend the knowledge concerning structural features of *S. bovis* and to investigate the structure of the new EPS of the *S. gallolyticus* subsp. *gallolyticus* strain DSM 13808 as an introduction to further research into *S. bovis* virulence factors and their possible correlation with CRC. 

## 2. Results

### 2.1. Isolation of S. gallolyticus *subsp.* gallolyticus DSM 13808 Exopolysaccharide

The EPS was isolated from bacterial cells (4.69 g) by trichloroacetic acid extraction, ethanol precipitation and then purified according to the method described by Gorska et al. [16], with a slight modification—ion exchange chromatography was not used. After DNAse, RNAse and protease treatment, EPS (46.5 mg) was purified by gel filtration on a HiLoad 16/600 Superdex 30 pg column. The first predominant fraction (3.6 mg) was used for further analysis. The average molecular weight of the EPS was estimated by gel permeation chromatography to be approximately 11.7 kDa.

### 2.2. Structure of the Exopolysaccharide

Sugar and methylation analyses of the EPS, together with determination of the absolute configuration, showed the presence of terminal d-Glc*p*, 2-substituted d-Glc*p,* 4-substituted d-Glc*p*, 3-substituted l-Rha*p* and 2,3-disubstituted l-Rha*p* in the ratio 1:1.8:0.6:0.7:0.3. Further analysis indicated the presence of 6-substituted d-Glc*p*, not observed in methylation analysis due to phosphate substitution.

**Figure 1:**
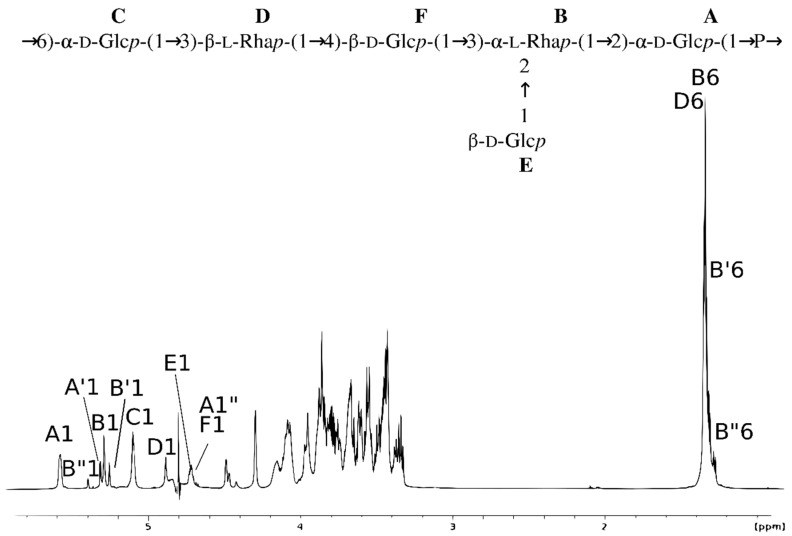
The structure and ^1^H NMR spectrum of the EPS isolated from *S. gallolyticus* DSM 13808. The capital letters refer to carbohydrate residues, as shown in the inset structure and Table 1. The Arabic numerals refer to protons in respective residues. Letter A′ presents a variant of the residue A due to the lack of a phosphate group at the reducing end, affecting the presence of residue B′ instead of residue B.

Preliminary one-dimensional (1D) ^1^H nuclear magnetic resonance (NMR) analysis of the EPS showed the presence of six major anomeric protons (A1-F1) and CH_3_ signals (B6, D6) characteristic of deoxysaccharides (Figure 1). 

**Figure 2:**
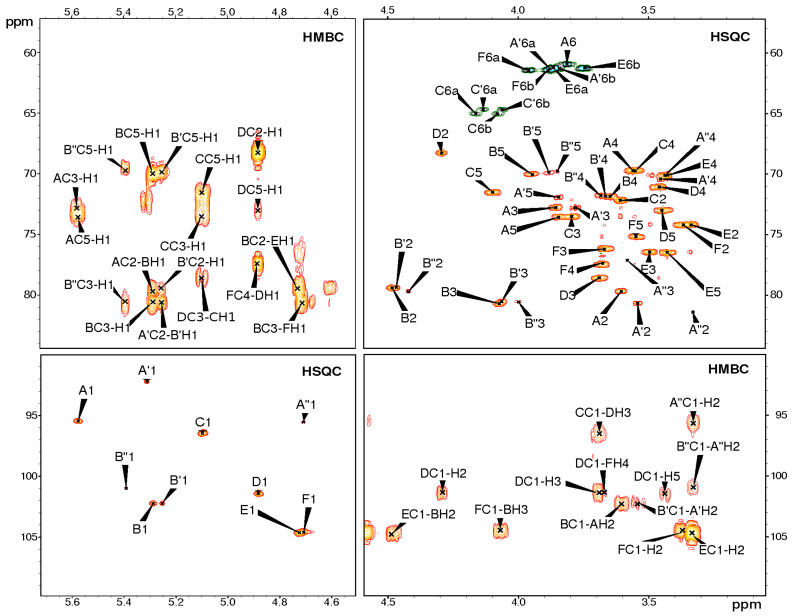
Selected regions of ^1^H,^13^C HSQC-DEPT and HMBC spectra of the EPS. The capital letters refer to carbohydrate residues, as shown in Figure 1 and Table 1. The Arabic numerals refer to protons and carbons in respective residues. Residues A′, A″ and residues B′, B″ are variants of residues A and B, respectively, due to the lack of a phosphate group at the reducing end.

The EPS was further analysed using two-dimensional (2D) ^1^H,^13^C-NMR spectroscopy. All the spin systems were assigned using a combination of COSY, TOCSY with different mixing times, HSQC-DEPT, HSQC-TOCSY, NOESY and HMBC spectra (Figure 2), and by comparison with previously reported NMR data for respective monosaccharides [17,18]. Chemical shift values and the identified inter-residue connectivities for the EPS are presented in Table 1.

The NMR analysis showed six major signals representing hexasaccharide repeating unit of EPS (residues A, B, C, D, E and F) (Figure 1 and Figure 2 and Table 1).

Residue A with the H1/C1 signals at δ 5.58/95.5 ppm, *J*_C-1,H-1_~173 Hz was recognised as the 2-substituted α-d-Glc*p* residue based on the downfield shift of the C2 signals (79.7 ppm) and the strong vicinal couplings between H2, H3, H4 and H5.

Residue B with the H1/C1 signals at δ 5.29/102.2 ppm, *J*_C-1,H-1_~176 Hz was assigned as the 2,3-disubstituted α-l-Rha*p* from the high chemical shifts of the C2 (79.4 ppm) and C3 (80.7 ppm) and the signals for an exocyclic CH_3_ group (1.33 ppm, 17.7 ppm). 

Residue C with the H1/C1 signals at δ 5.10/96.5 (*J*_C-1,H-1_~170 Hz) was recognized as the 6-substituted α-d-Glc*p* based on the large vicinal couplings between all ring protons and the characteristic downfield shift of the C6 signal (64.9 ppm).

Residue D with the H1/C1 signals at δ 4.88/101.4 (*J*_C-1,H-1_~162 Hz) was recognized as the 3-substituted β-l-Rha*p* based on the high chemical shifts of the C-3 (78.6 ppm) and the characteristic shift for an exocyclic CH_3_ group (1.34 ppm, 17.6 ppm). 

Residue E with the H1/C1 signals at δ 4.72/104.7 (*J*_C-1,H-1_~161 Hz) was assigned as terminal β-d-Glc*p* according to coupling constants between all protons in the sugar ring.

Residue F with the H1/C1 signals at δ 4.71/104.5 (*J*_C-1,H-1_~163 Hz) was assigned as 4-substituted β-d-Glc*p* according to downfield shift of the C-4 at δ 77.4 ppm. 

**Figure 3:**
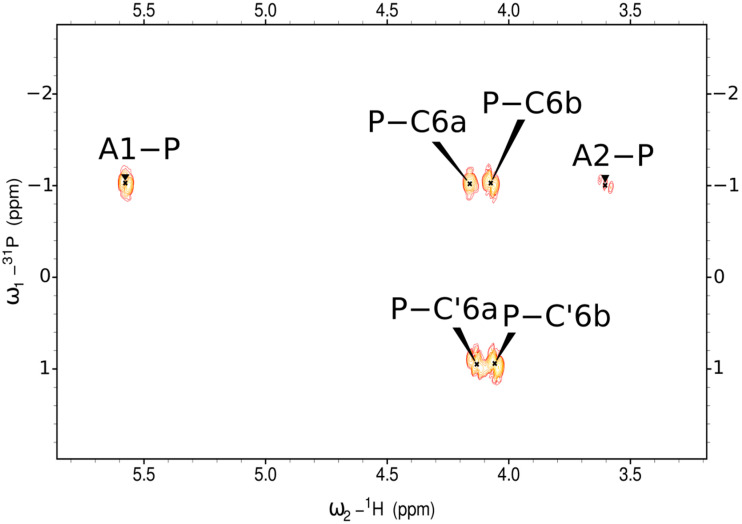
Selected region of ^1^H,^31^P HMBC spectrum of the EPS.

The HMBC ^1^H-^31^P NMR spectrum (Figure 3) of the EPS showed the presence of the glycosidically linked phosphate group (P). The ^1^H-^31^P correlations between the ^31^P signal at δ_P_ −1.02 ppm and H1 of A (2-substituted α-d-Glc*p*) and H6a and H6b of C (6-substituted α-d-Glc*p*) indicated the phosphodiester group. The ^31^P signal at δ_P_ 0.95 ppm showed connectivity to H6a and H6b of residue C′, which is a variant of C residue, indicating phosphomonoester at the non-reducing end of the EPS.

Residues A′ (H1/C1 signals at δ 5.31/92.2 ppm) and A″ (H1/C1 signals at δ 4.71/95.6 ppm) were recognized as α and β variants of residue A (2-substituted α-d-Glc*p*-(1→P) devoid of the phosphate group at the reducing end of the EPS, which affected adjacent residues B′ (H1/C1 signals at δ 5.26/102.2 ppm) and B″ (H1/C1 signals at δ 5.39/101.0 ppm), recognized as variants of residue B (2,3-disubstituted α-l-Rha).

The sequence of sugar residues in the EPS was identified by HMBC (Figure 2, Table 1) and NOESY (Table 1) experiments; the revealed structure is presented in Figure 1.

### 2.3. Mass Spectrometry Analysis of the EPS

**Figure 4:**
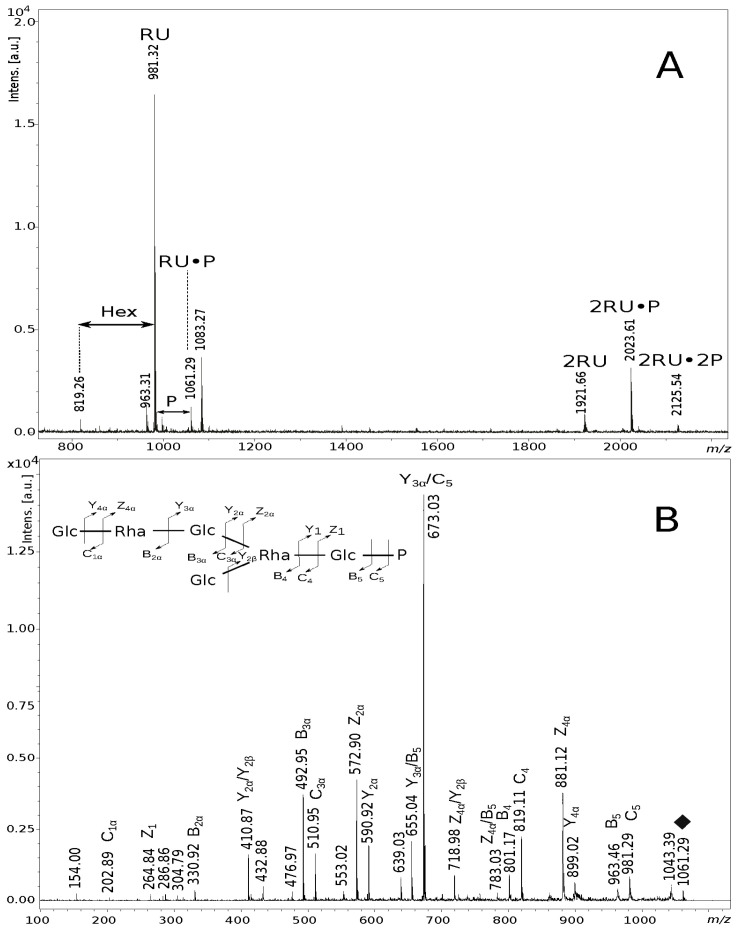
Positive ion mode MALDI-TOF mass spectra of the partially hydrolysed EPS (**A**), and the MS/MS fragmentation of the ion at *m*/*z* 1061.29 (1+) attributed to the hexasaccharide repeating unit linked to phosphate group accompanied by the EPS inset structure explaining interpretation of the fragment ions (**B**). The RU·P and RU symbols stand for the one repeating unit of the EPS with or without P, respectively. The *m*/*z* values represent single charged ions. The fragment ions were presented according to the nomenclature of Domon and Costello [19].

The elucidated structure of the EPS isolated from *S. gallolyticus* DSM 13808 was confirmed by matrix-assisted laser-desorption/ionisation time-of-flight (MALDI-TOF) mass spectrometry. The mass spectrum of the partially hydrolysed EPS (Figure 4A) showed ions at *m*/*z* 981.32 [M + H, Na]^+^ and *m*/*z* 963.31 [M–H_2_O + H, Na]^+^. These ions correspond to the repeating unit (RU) built of four Glc and two Rha molecules, which, together, give a calculated monoisotopic mass of 958.34 Da. Ions at *m*/*z* 1061.29 [M + H, Na]^+^ and *m*/*z* 1083.27 [M + H, 2Na]^+^ represent four Glc and two Rha residues, and, additionally, one phosphate group as a compound of the repeating unit. Furthermore, the less abundant ions at *m*/*z* 1921.66 [M + H, Na]^+^, *m*/*z* 2023.61 [M + H, 2Na]^+^ and *m*/*z* 2125.54 [M + H, 3Na]^+^ indicated structures built of two RUs, two RUs with one phosphate group and two RUs with two phosphate group, respectively.

Additionally, the presence and sequence of sugars and location of the phosphate group (P) in the EPS was indicated by MALDI-TOF tandem mass spectrometry (MS/MS) analysis of the ion at *m*/*z* 1061.29 (Figure 4). The MS/MS fragmentation spectrum consisted of ions at *m*/*z* 264.84 [Glc·P, Z_1_], *m*/*z* 410.87 [Rha·Glc·P, Y_2α_/Y_2β_], *m*/*z* 572.90 [Glc·Rha·Glc·P, Z_2α_], *m*/*z* 718.98 [Rha·Glc·Rha·Glc·P, Z_4α_/Y_2β_] and *m*/*z* 881.12 [Glc·Rha·Glc·Rha·Glc·P, Z_4α_], indicated locations of P at the Glc residue and supported the sequence of the identified monosaccharides. An interpretation of the observed ions with mass spectra is shown in Table 2.

## 3. Discussion

Exopolysaccharides play an important role as components of biofilm involved in cell adhesion to surfaces. These macromolecules protect microorganisms against mechanical damage and dehydration but also against external environment stressors, such as temperature, pH, salinity, pressure, heavy metals, radiation and a host’s immune defence and biological factors, e.g., bacteriocins, bacteriophages and antibiotics. Bacterial EPSs exhibit various properties, with commercial applications in the medicine, pharmaceutical and food industries, agriculture and soil remediation. It is worth noting that biomedical properties of bacterial EPSs, e.g., *Paenibacillus polymyxa* curdlan sulphate, allow for using it as a vaccine against infection of hepatitis B virus and treatment of severe malaria [20]. *Lactobacillus plantarum* 70810 EPS inhibits proliferation of tumour cells, such as HepG-2, BGC-823 and HT-29 [21]. *Nostoc* sp. EPS was used as an aerogel for drug delivery and tissue engineering [22]. Exopolysaccharides of probiotic bacteria have immunomodulatory activities, providing health benefits to the host [23,24,25]. Due to these applications, chemical, physicochemical and biological studies of EPSs are very important subjects.

Little is known about biofilm of Sgg that may contribute to chronic inflammation and the CRC development. It is known that biofilms are surface-associated communities of bacteria that are embedded in a hydrated matrix of extracellular polymeric substances. Biofilms pose a substantial health risk and are key contributors to many chronic and recurrent infections. This link between biofilm-associated bacteria (e.g., *Helicobacter pylori*) and chronic infections likely arises from an increased tolerance to conventional antibiotic treatments as well as immune system activity. One of the major sugar antigens loosely attached to the cell surface is EPS. Polysaccharide capsule, piluses and the type VII secretion system were indicated as the main determinants of Sgg-specific association with endocarditis and CRC [11,12,13]. 

Exopolysaccharides of *S. bovis* are poorly characterized molecules. Until now, only the single *S. macedonicus* Sc136 EPS [15] structure was known among the entire *S. bovis* group. The *S. macedonicus* Sc136 strain, found in Greek sheep and goat cheeses, produces a high-molecular-mass, highly texturizing EPS built of hexasaccharide repeating units consisting of D-glucose, D-galactose and N-acetyl-D-glucosamine [15]. 

In this study, we have determined the structure of the EPS produced by *S. gallolyticus* subsp. *gallolyticus* for the first time. The repeating unit building the Sgg DSM 13808 EPS was elucidated as the hexasaccharide composed of the four d-Glc and two l-Rha residues and phosphate group (Figure 1). These residues are common constituents found in sugar surface antigens. The Glc and Rha residues, together with the phosphate group, have been previously identified as components (e.g., the *S. pneumoniae* 16F capsular polysaccharide [18] and the *Lactobacillus sake* 0-1 EPS [26]) but to differ in sequence and sugar substitution. 

An interesting feature of Sgg 13808 EPS is the presence of a phosphate group at the reducing end of the EPS, identified before, e.g., for *Hafnia alvei* PCM 1195 O-antigen [27] and capsular polysaccharide of *Pasteurella haemolytica* serotype A7 [28]. Phosphate groups are essential for conformation of the entire EPS molecule as carriers of negative charges, playing an important role stabilizing the bacterial cell surface by interacting with doubly charged Ca^2+^ and Mg^2+^ ions.

Presented herein for the first time, the EPS structure of *S. gallolyticus* subsp. g*allolyticys* DSM 13808 broadens the knowledge regarding *S. bovis* exopolysaccharides and may help to understand the importance of bacterial surface structures factors, including sugar antigens, in the aetiology and pathogenesis of infectious diseases and hypothesized connection with CRC.

## 4. Materials and Methods

### 4.1. Bacteria

*Streptococcus gallolyticus* subsp. *gallolyticus* DSM 13808 (previously known as *S. bovis* biotype I) was isolated from anaerobic digester fed with shea cake and was obtained from the German Collection of Microorganisms and Cell Cultures GmbH (Brunswick, Germany). Bacteria were grown for 24 h in brain–heart infusion broth (10 l) at 37 °C. 

### 4.2. Exopolysaccharide

The EPS was isolated and purified as previously described [16] with the following modification. Lyophilized bacteria (4.69 g) were extracted with 10% TCA (25 °C, 2.5 h) and then centrifuged (13,000× *g*, 4 °C, 20 min). EPS was precipitated from obtained supernatant with five volumes of cold 96% ethanol (4 °C, overnight) and finally centrifuged (13,500× *g*, 20 min). The pellet suspended in water was dialysed extensively for 48 h against water and then freeze-dried. Crude EPS dissolved in buffer (50 mM Tris–HCl pH 7.5, 10 mM MgCl_2_) was treated with DNase and RNase (37 °C, 6 h) and with protease from *Streptomyces griseus* (37 °C, overnight, SIGMA), then dialysed against water at 4 °C for 24 h. The EPS was purified by size-exclusion chromatography on a HiLoad Superdex 30 pg column (1.6 × 60 cm) equilibrated with 0.05 M acetic acid. A Shodex (RI 102) differential refractometer (Showa Denko Europe GmbH, Munich, Germany) was used for monitoring of eluates. After lyophilisation, first predominant fraction (3.6 mg) was used for further analysis.

### 4.3. Partial Acid Hydrolysis

A sample of the EPS (0.2 mg) was hydrolysed with 48% HF (0.2 mL) at −20 °C for 24 h and checked by MALDI-TOF MS after lyophilisation.

### 4.4. Analytical Methods

Monosaccharides were determined as their alditol acetates by GC–MS [29]. Partially methylated alditol acetates were examined as described by Ciucanu and Kerek [30] by GC–MS. Thermo Scientific ITQ system using a ZebronTM ZB-5HT (Thermo Fisher Scientific, Waltham, MA, USA) GC Capillary Column (30 m × 0.25 mm × 0.25 μm) was used with temperature program gradient from 150 to 270 °C at 8 °C/min. The absolute configurations were determined using (−)-2-butanol for the formation of 2-butyl glycosides [31,32]. The average molecular mass of the EPS was measured by gel permeation chromatography on an OHpak SB-806M HQ column (8 × 300 mm, Shodex, Showa Denko Europe GmbH, Munich, Germany) calibrated with dextran standards (Mw 1, 5, 12, 25, 50, 80, 150 and 270 kDa) and equilibrated with 0.1 M ammonium acetate buffer. The column eluate was monitored with a refractive index detector (RI 102; Shodex).

### 4.5. NMR Spectroscopy

NMR spectra were recorded with Avance III 600 MHz spectrometer (Bruker BioSpin, Rheinstetten, Germany) using 5 mm QCI cryoprobe; 3 mm tubes (~160 μL). The EPS sample was three times repeatedly exchanged with D_2_O (99%) and freeze-dried. NMR spectra were obtained of D_2_O solutions at 25 °C. Internal reference was applied using acetone (2.225 ppm, 31.05 ppm). The NMR signals were assigned by set of experiments (COSY, TOCSY, NOESY, HMBC, HSQC-TOCSY and HSQC-DEPT) using the NMRFAM-SPARKY program [33]. The *J*_C-1,H-1_ constant values were achieved from a non-decoupled HSQC-DEPT experiment. The TOCSY experiments were run with the mixing times 30, 60 and 100 ms. The delay time in the HMBC experiment was set to 60 ms, and the mixing time in the NOESY experiment was 200 ms. ^1^H,^31^P HMBC NMR spectrum was recorded for observation of phosphate groups by using Standard Bruker software (Bruker BioSpin GmbH, Rheinstetten, Germany), used for acquiring and processing.

### 4.6. Mass Spectrometry

The MALDI-TOF mass spectra were obtained in a positive ion mode by using an Ultraflextreme (Bruker, Bremen, Germany) mass spectrometer. Samples were dissolved in water (1 mg/mL). The 2,5-Dihydroxybenzoic acid (10 mg/mL in 1:1 AcN/0.2 M citric acid [*v*/*v*]) was used as a matrix. MALDI TOF/TOF MS analysis was performed by LIFT mode with the ion source voltage of 7.5 kV. The precursor ion mass window was set at 4 Da. The precursor ion was accelerated at 19.0 kV in the LIFT cell. The reflector voltage was set at 29.5 kV. External calibration was obtained with the Peptide Calibration Standard II (Bruker Daltonics, Germany). Ions were described according to the nomenclature of Domon and Costello [19].

## Figures and Tables

**Table 1:** The ^1^H and ^13^C NMR chemical shifts and selected inter-residue correlations from NOESY and HMBC spectra of the EPS isolated from *S. gallolyticus* DSM 13808.

Residue ^(a)^		Chemical Shifts (ppm)						Selected Inter-Residue NOE and ^3^*J*_H,C_ Connectivities	
		H1 C1	H2, C2	H3 C3	H4 C4	H5 C5	H6a, H6b C6	H1/C1 Connectivities to	Inter-Residue Atom/Residue
A	→2)-α-d-Glc*p*-(1→P ^(b)^	5.58 95.5	3.61 79.7	3.86 72.8	3.55 69.7	3.85 73.6	3.81 ^(c)^ 61.0	-	-
A′	→2)-α-d-Glc*p*-(1→	5.31 92.2	3.54 80.7	3.78 72.8	3.45 70.4	3.85 71.8	3.88, 3.84 61.3	-	-
A″	→2)-β-d-Glc*p*-(1→	4.71 95.6	3.33 81.3	3.58 77.1	3.44 70.1	nd ^(d)^	nd ^(d)^	-	-
B	→2,3)-α-l-Rha*p*-(1→	5.29 102.2	4.49 79.4	4.07 80.7	3.65 71.9	3.95 70.0	1.33 17.7	3.61 79.7	H2 of A
B′	→2,3)-α-l-Rha*p*-(1→	5.26 102.2	4.47 79.4	4.07 80.5	3.67 71.8	3.88 69.9	1.31 17.4	3.54 80.7	H2 of A′
B″	→2,3)-α-l-Rha*p*-(1→	5.39 101.0	4.42 79.6	4.00 80.5	3.68 71.8	3.85 69.7	1.28 17.4	3.33 81.3	H2 of A″
C	P→6)-α-d-Glc*p*-(1→	5.10 96.5	3.61 72.2	3.80 73.5	3.55 69.7	4.10 71.5	4.16, 4.08 64.9	3.69 78.6	H3 of D
D	→3)-β-l-Rha*p*-(1→	4.88 101.4	4.30 68.3	3.69 78.6	3.46 71.1	3.45 73.0	1.34 17.6	3.68 77.4	H4 of F
E	β-d-Glc*p*-(1→	4.72 104.7	3.34 74.2	3.50 76.4	3.43 70.1	3.43 76.5	3.87, 3.75 61.2	4.49 79.4	H2 of B
F	→4)-β-d-Glc*p*-(1→	4.71 104.5	3.37 74.2	3.67 76.2	3.68 77.4	3.55 75.2	3.96, 3.89 61.4	4.07 80.7	H3 of B

^(a^^)^ The *J*_C-1,H-1_ constants 173, 172, 162, 176, 174, 173, 170, 162, 161 and 163 Hz were observed for A, A′, A″, B, B″, C, D, E and F residues, respectively. ^(b^^)^ The ^31^P resonance at δ_P_ −1.02 ppm showed connectivity to the H1 of residue A and H6a; H6b of residue C indicated the phosphodiester group. Residues A′ and A″ were recognized as α and β variants of residue A due to the lack of a phosphate group at the reducing end of the EPS, which affected the adjacent residues B′ and B″, recognized as variants of residue B. The ^31^P signal at δ 0.95 ppm showed connectivity only to H6a and H6b of residue C′, indicating phosphomonoester at the non-reducing end of the EPS. ^(c)^ Not resolved. ^(d)^ Not determined.

**Table 2:** Interpretation of positive ion mode MALDI-TOF mass spectra of the partially hydrolysed EPS (Figure 4A) and the MS/MS fragmentation of the hexasaccharide represented by the ion at *m*/*z* 1061.29 (inset structure) (Figure 4B).

Oligosaccharide Structure	Calculated Mass (Da)	Observed Ion (*m*/*z*)	Calculated Ion (*m*/*z*)	Interpretation of the Ion
Glc_8_·Rha_4_·P_2_	2058.60	2125.54	2125.55	[M + H, 3Na]^+^
Glc_8_·Rha_4_·P	1978.63	2023.61	2023.60	[M + H, 2Na]^+^
Glc_8_·Rha_4_	1898.66	1921.66	1921.65	[M + H, Na]^+^
Glc_4_·Rha_2_·P	1038.30	1083.27	1083.28	[M + H, 2Na]^+^
Glc_4_·Rha_2_·P	1038.30	1061.29	1061.29	[M + H, Na]^+^
Glc_4_·Rha_2_·P	1038.30	1043.39	1043.28	[M–H_2_O + H, Na]^+^
Glc_4_·Rha_2_	958.34	981.29	981.33	[M + H, Na]^+^
Glc_4_·Rha_2_	958.34	963.46	963.31	[M–H_2_O + H, Na]^+^
Glc_3_·Rha_2_·P	876.25	899.02	899.24	[M + H, Na]^+^
Glc_3_·Rha_2_·P	876.25	881.12	881.23	[M–H_2_O + H, Na]^+^
Glc_3_·Rha_2_	796.28	819.11	819.27	[M + H, Na]^+^
Glc_3_·Rha_2_	796.28	801.17	801.26	[M–H_2_O + H, Na]^+^
Glc_3_·Rha_2_	796.28	783.03	783.26	[M–2H_2_O + H, Na]^+^
Glc_2_·Rha_2_·P	714.20	718.98	719.18	[M–H_2_O + H, Na]^+^
Glc_3_·Rha	650.23	673.07	673.22	[M + H, Na]^+^
Glc_3_·Rha	650.23	655.04	655.20	[M–H_2_O + H, Na]^+^
Glc_2_·Rha·P	568.14	590.92	591.13	[M + H, Na]^+^
Glc_2_·Rha·P	568.14	572.90	573.12	[M–H_2_O + H, Na]^+^
Glc_2_·Rha	488.17	510.95	511.16	[M + H, Na]^+^
Glc_2_·Rha	488.17	492.95	493.15	[M–H_2_O + H, Na]^+^
Glc·Rha·P	406.09	432.88	433.05	[M–H_2_O + H, 2Na]^+^
Glc·Rha·P	406.09	410.87	411.06	[M–H_2_O + H, Na]^+^
Glc·Rha	309.12	330.92	331.11	[M + H, Na]^+^
Glc·P	242.02	304.79	305.00	[M + H, 2Na]^+^
Glc·P	242.02	286.86	287.01	[M–H_2_O + H, 2Na]^+^
Glc·P	242.02	264.84	265.02	[M + H, Na]^+^
Glc	180.06	202.97	203.05	[M + H, Na]^+^

## Data Availability

The data presented in this study are available on request from the corresponding author.

## Abbreviations

EPS	exopolysaccharide
TCA	trichloroacetic acid
MALDI-TOF	matrix-assisted laser-desorption/ionisation time-of-flight
MS	mass spectrometry
NMR	nuclear magnetic resonance
COSY	correlated spectroscopy
TOCSY	total correlation spectroscopy
NOESY	nuclear Overhauser effect spectroscopy
HMBC	heteronuclear multiple bond correlation
HSQC	heteronuclear single quantum coherence
DEPT	distortionless enhancement by polarisation transfer
CRC	colorectal cancer
DSMZ	German Collection of Microorganisms and Cell Cultures GmbH
